# Aroma Profile Development in Beer Fermented with Azacca, Idaho-7, and Sultana Hops

**DOI:** 10.3390/molecules28155802

**Published:** 2023-08-01

**Authors:** Anna K. McCabe, Jasmine K. Keyes, Heidi Hemetsberger, Chris V. Kurr, Bryan Albright, Michael G. Ward, Megan L. McKinley, Steven J. Breezley, Callie A. Cole

**Affiliations:** 1 Department of Chemistry & Biochemistry, Fort Lewis College, 1000 Rim Drive, Durango, CO 81301, USA; kkmccabe@fortlewis.edu (A.K.M.); jkkeyes@fortlewis.edu (J.K.K.); mgward@fortlewis.edu (M.G.W.); mlmckinley@fortlewis.edu (M.L.M.); 2 Ska Brewing Company, 225 Girard St., Durango, CO 81303, USA; heidi@skabrewing.com (H.H.); kurr@skabrewing.com (C.V.K.); balbright@skabrewing.com (B.A.); breeze@skabrewing.com (S.J.B.)

**Keywords:** hops, aroma, gas chromatography, solid phase microextraction, biotransformation

## Abstract

Hops are among the most costly and environmentally impactful raw materials used in brewing, yet they play a crucial role in the aroma of beer. However, predicting beer aroma based on hop variety or hopping method remains arduous. This is partly because hop oils are unique for each hop variety, and they may be biotransformed by yeast enzymes during fermentation. Even slight molecular structure modifications can dramatically affect the organoleptic properties of beer. Through combined chemical and sensory analysis of dry-hopped beers prepared with different hop varieties (Azacca, Idaho-7, and Sultana), this work aimed to profile the aromas and the overall biotransformation processes taking place during fermentation. A total of 51 volatile organic compounds (VOCs) were semi-quantified and monitored: 19 esters, 13 sesquiterpenes, 7 ketones, 7 alcohols, 4 monoterpenes, and 1 volatile acid. There were significant similarities in the measured analytes and perceived aromas of these beers, but one hop variety (Sultana) delivered an increased quantity of unique aromas and an increased concentration of volatiles in the headspace for the same quantity of hop pellets added. This work provides practical information to brewers who utilize hops in beer production.

## 1. Introduction

**Figure 1:**
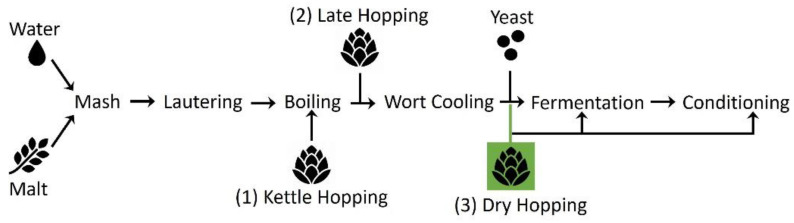
This brewing process flow chart includes the 3 most common hopping methods: (1) kettle hopping, (2) late hopping, and (3) dry hopping. In this experiment, hops are added at the same time as inoculation (green box).

Hops (*Humulus lupulus* L.) have been implemented for a wide range of medicinal purposes since prehistoric times [1]. In recent years, the utilization of ever diverse hop varieties and hopping methods in the brewing process has gained substantial popularity. In 2021, the value of U.S. hops production was $662 million, which was 7% higher than the year prior [2]. Hop use has increased despite the fact that it is an expensive raw ingredient from both a financial and environmental standpoint because it imparts unique flavor and aroma profiles to beer that are imperative for customer satisfaction [3]. Previous work has shown that the choice of hop variety as well as the method of hop addition are of great importance to the quality of the final product [4,5,6]. Several recent publications have also compared the organoleptic qualities resulting from specific hop varieties and dry hopping methods [7,8,9,10]. However, few previous studies have surveyed the chemical and sensory attributes of Azacca- [11,12], Idaho-7-, and Sultana-hopped beers. Sultana is of particular interest because it is a newly released variety [13]. Notably, the hoppy aroma of dry-hopped beer is often a result of complex chemical and biological processes following the extraction of hop-derived oils into the beer. Yeast enzymes can catalyze the biotransformation of hop oil compounds, which may be more conjugated and often odorless in the raw hops, into more aroma-active compounds yielding desirable organoleptic properties in the final beer [3]. It is difficult to predict the impact of hops on the aroma of the final beer due to these biotransformations, which still require further study [3]. This challenge has led to increased interest in not only the aroma profiles of beers hopped with various hop varieties but in the yeast biotransformation processes that yield more aroma-active species from their hop oils [3]. For this reason, at Ska Brewing Co. in Durango, CO, USA, hop addition and yeast inoculation occur simultaneously to increase the amount of time that the yeast and hops are in direct contact, thereby promoting biotransformation. After inoculation and hopping, careful monitoring of aroma species throughout the fermentation process can lend valuable insights into the biotransformations that take place with different hop varieties [14]. A flow chart of the brewing process and the three most common hopping methods as well as the dry hopping method used in this work are shown in Figure 1. 

Only through the study of different hop varieties and the extraction and biotransformation of their oils can the brewing process involving these raw materials be optimized. Herein, we performed both chemical and sensory analysis of single-hopped beer prepared at Ska Brewing Co. with three hop varieties: Azacca, Idaho-7, and Sultana. Employing previously optimized methods for the profiling of volatile organic compounds (VOCs) using headspace-solid phase microextraction–gas chromatography–mass spectrometry (HS-SPME-GC-MS) [15,16], we identified the major esters, ketones, alcohols, acids, monoterpenes, and sesquiterpenes and monitored their concentrations over time as fermentation progressed. Lastly, we conducted a sensory panel to compare perceived aroma and measured VOCs. It is well known that the concentration of VOCs and their precursors in hopped beer depends on many factors, including the variety of hops used, the timing of hop addition, and the biotransformations of the hop oils that result from that timing. The central aim of this work was to explore the differences in aroma and the overall biotransformation processes taking place during the fermentation of three single-hopped beers through both chemical and sensory means.

## 2. Results and Discussion

### 2.1. Comparison of Hop Varieties (Azacca, Idaho-7, and Sultana)

**Figure 2:**
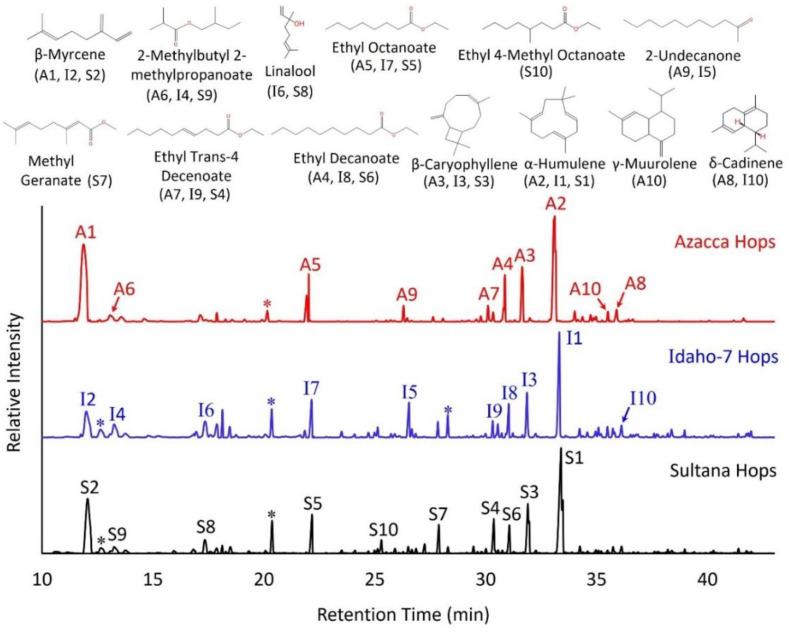
Three chromatograms showing the top ten VOCs (in order of abundance) detected in the final beer headspace after hopping with Azacca hops (red, labelled A1–A10), Idaho-7 hops (blue, labelled I1–I10), and Sultana hops (black, labelled S1–S10). Triplicate chromatograms were averaged, and the control (no hops) chromatogram was subtracted, meaning every VOC shown here is a result of hop addition. See Table 2, Table 3 and Table 4 for detailed semiquantification results. Peaks labelled * are siloxanes originating from the GC column or SPME fiber [22,23].

The chromatograms labelled with the top ten most abundant VOCs measured in the final, single-hopped beer samples prepared with Azacca hops (red), Idaho-7 hops (blue), and Sultana hops (black) are included in Figure 2. Notably, many significant aroma compounds were measured at low concentrations, and therefore do not stand out in Figure 2. These species are discussed in detail in the following section and included in Table 1, Table 2 and Table 3. As Figure 2 illustrates, there were many similarities in the most abundant VOCs measured across these three hop varieties. Seven of the top ten analytes were shared across all three single-hopped beers, although they varied in abundance (β-myrcene, 2-methylbutyl 2-methylpropoanoate, ethyl octanoate, ethyl trans-4-decenoate, ethyl decanoate, β-caryophyllene, and α-humulene). According to published odor descriptions of these analytes, the ethyl esters create a fruity, pineapple, pear, and brandy aroma while the monoterpenes and sesquiterpenes add woody, spicy, herbal notes at specific perception thresholds [17,18,19,20]. The aroma of 2-methylbutyl 2-methyl propanoate has been described as ‘earthy’ [19]. Sensory analyses, which we include in a later section, are important to elucidate which VOCs dominate the overall perceived aroma based on their concentration in the headspace and their odor perception thresholds.

Hopping beer with Sultana resulted in a headspace with not only the greatest number of unique volatiles (39) but also an overall higher semi-quantified concentration (3.2 mg L^–1^) than beers hopped with Azacca (1.7 mg L^–1^) or Idaho-7 (0.6 mg L^–1^). Of the top ten analytes shown in Figure 2, two are unique to Sultana-hopped beer: ethyl 4-methyl octanoate and methyl geranate. Methyl geranate aroma has been described as ‘sweet, candy’ [21], and although ethyl 4-methyl octanoate does not have a published odor description, it is chemically very similar to other ethyl esters with fruity aromas. (It should be noted that methyl geranate was measured in both Azacca- and Idaho-7-hopped beer but at an order of magnitude lower abundance than Sultana-hopped beer).

Idaho-7 and Azacca hops yielded many similar VOCs. 2-Undecanone (orange) [24] and δ-cadinene (woody) [18] were abundant in both Azacca- and Idaho-7-hopped beer. Linalool (sweet, floral, orange) [19] and γ-muurolene were measured in all hop varieties, but only made the top ten in Idaho-7 and Sultana, and Azacca, respectively. Although the most abundant analytes certainly shape the aroma of hopped beer when they exceed perception thresholds, it is important to look at less abundant analytes that may have a lower olfactory detection threshold as well. Table 1, Table 2 and Table 3 include the semi-quantified concentrations of every analyte measured in this study. 

**Table 1:** Semi-quantified **ester concentrations** (mg/L) ^1^ measured in beer headspace at various stages after simultaneous inoculation/hopping (6 h, 24 h, final) for three hop varieties (Azacca, Idaho-7, and Sultana).

			Azacca Hops			Idaho-7 Hops			Sultana Hops			
Family	Analyte	RT ^2^	6 h	24 h	Final	6 h	24 h	Final	6 h	24 h	Final	Aroma
Methyl ester	Methyl 4-ethyl 4-pentenoate	13.6	0.050 ± 0.011	0.040 ± 0.002		0.015 ± 0.004						Sweet, yeast [19]
	Methyl 6-methyl heptanoate	16.6	0.018 ± 0.004			0.004 ± 0.001						
	Methyl 6-methyl octanoate	21.7							0.013 ± 0.004	0.025 ± 0.008		
	Methyl 4-decenoate	27.2	0.028 ± 0.007	0.004 ± 0.001		0.024 ± 0.007	0.041 ± 0.011		0.061 ± 0.018	0.202 ± 0.061	0.040 ± 0.016	
	Methyl geranate	27.7	0.008 ± 0.001	0.008 ± 0.002	0.011 ± 0.003	0.009 ± 0.004	0.018 ± 0.007	0.013 ± 0.004	0.031 ± 0.009	0.192 ± 0.058	0.116 ± 0.021	Sweet, candy [21]
	Methyl 3,6-dodecanoate	35.4							0.013 ± 0.004	0.018 ± 0.006		
Ethyl ester	Ethyl heptanoate	17.1			0.013 ± 0.003						0.019 ± 0.001	Fruity [25]
	Ethyl octanoate	22.0	0.012 ± 0.004	0.041 ± 0.012	0.088 ± 0.022			0.025 ± 0.009	0.004 ± 0.001	0.173 ± 0.052	0.160 ± 0.030	Fruity, brandy [20]
	Ethyl phenylethanoate	24.0			0.006 ± 0.001							
	Ethyl 4-methyl octanoate	25.1								0.032 ± 0.010	0.056 ± 0.005	
	Ethyl nonanoate	26.5		0.007 ± 0.001	0.012 ± 0.003			0.010 ± 0.003		0.023 ± 0.007	0.016 ± 0.001	Fruity [20]
	Ethyl 8-methyl nonanoate	29.2			0.005 ± 0.001					0.033 ± 0.010	0.027 ± 0.005	
	Ethyl trans-4-decenoate	30.1		0.008 ± 0.001	0.034 ± 0.008			0.019 ± 0.006		0.073 ± 0.022	0.160 ± 0.012	Pear, pineapple [17]
	Ethyl decanoate	30.9		0.017 ± 0.003	0.096 ± 0.024			0.024 ± 0.008		0.117 ± 0.035	0.123 ± 0.017	Fruity [20]
	Ethyl dodecanoate	38.8						0.003 ± 0.001		0.031 ± 0.009	0.014 ± 0.002	Floral, fruity [20]
Other ester	1-methylbutyl propionate	11.0	0.028 ± 0.008			0.028 ± 0.001	0.011 ± 0.004					
	2-methylbutyl 2-methylpropanoate	13.1	0.086 ± 0.020	0.070 ± 0.017	0.039 ± 0.010	0.036 ± 0.010	0.077 ± 0.031	0.043 ± 0.015	0.024 ± 0.007	0.087 ± 0.026	0.076 ± 0.008	Earthy [19]
	Terpinyl acetate ^3^	13.5							0.017 ± 0.005	0.069 ± 0.021		Spice, herbal, citrus [26]
	2-ethylhexyl penatanoate	17.4									0.014 ± 0.001	

^1^ All results are reported as $\bar{x}$ ± s for triplicate experiments. Control (non-hopped) results were subtracted from these data. From the 6 h sample to the final sample, analytes in red decreased in concentration and analytes in green increased in concentration. Analytes in black did not consistently increase or decrease in all samples. ^2^ Retention time (RT) is reported in min. ^3^ Analyte matched <10% with the NIST database.

**Table 2:** Semi-quantified **ketone, alcohol, and volatile acid concentrations** (mg/L) ^1^ measured in beer headspace at various stages after simultaneous inoculation/hopping (6 h, 24 h, final) for three hop varieties (Azacca, Idaho-7, and Sultana).

			Azacca Hops			Idaho-7 Hops			Sultana Hops			
Family	Analyte	RT ^2^	6 h	24 h	Final	6 h	24 h	Final	6 h	24 h	Final	Aroma
Ketone	2-Nonanone	16.8		0.020 ± 0.003	0.010 ± 0.003		0.019 ± 0.008	0.013 ± 0.004				Fruity [24]
	2-Decanone	21.6	0.011 ± 0.003	0.011 ± 0.001	0.008 ± 0.002	0.005 ± 0.001	0.014 ± 0.006	0.009 ± 0.003		0.024 ± 0.007	0.014 ± 0.003	Citrusy, orange [27]
	6-Undecen-2-one	25.5	0.007 ± 0.002	0.006 ± 0.001	0.004 ± 0.001							
	2-Undecanone	26.3	0.024 ± 0.006	0.026 ± 0.002	0.032 ± 0.008	0.024 ± 0.010	0.025 ± 0.010	0.041 ± 0.014		0.044 ± 0.013	0.032 ± 0.005	Orange [24]
	Jasmine lactone	30.6						0.013 ± 0.004			0.013 ± 0.002	Fruity, sweet, floral [28]
	2-Dodecanone	30.7	0.004 ± 0.001	0.003 ± 0.001			0.008 ± 0.003	0.004 ± 0.001			0.006 ± 0.002	Fruity, citrus [29]
	2-Tridecanone	34.9		0.007 ± 0.002	0.008 ± 0.002	0.009 ± 0.004	0.037 ± 0.015	0.009 ± 0.003	0.003 ± 0.001	0.021 ± 0.006	0.011 ± 0.002	Fruity, green [30]
Alcohol	Isothujol ^3^	13.7									0.033 ± 0.001	
	α-Terpineol ^3^	13.8					0.018 ± 0.007	0.013 ± 0.004				Lilac [24]
	Linalool	17.1	0.025 ± 0.002	0.037 ± 0.011	0.012 ± 0.003	0.029 ± 0.002	0.027 ± 0.011	0.038 ± 0.013	0.018 ± 0.005	0.026 ± 0.008	0.101 ± 0.007	Sweet, floral, orange [19]
	Citronellol	23.5						0.007 ± 0.002			0.014 ± 0.003	Sweet, floral [24]
	cis-Geraniol	24.7				0.009 ± 0.002					0.015 ± 0.005	Orange, citrus [19]
	2-Undecanol	26.7			0.003 ± 0.001			0.003 ± 0.001	0.005 ± 0.001	0.021 ± 0.006	0.019 ± 0.004	Fruity [24]
	Humuleneol II	40.1		0.003 ± 0.001	0.007 ± 0.002				0.004 ± 0.001	0.015 ± 0.005	0.011 ± 0.003	Floral, spicy, citrus [31]
Voltaile acid	Gamolenic acid ^3^	41.2							0.004 ± 0.001	0.022 ± 0.007	0.017 ± 0.003	

^1^ All results are reported as $\bar{x}$ ± s for triplicate experiments. Control (non-hopped) results were subtracted from these data. From the 6 h sample to the final sample, analytes in green increased in concentration. Analytes in black did not consistently increase or decrease in all samples. ^2^ Retention time (RT) is reported in min. ^3^ Analyte matched <10% with the NIST database.

**Table 3:** Semi-quantified **monoterpene and sesquiterpene concentrations** (mg/L) ^1^ measured in beer headspace at various stages after simultaneous inoculation/hopping (6 h, 24 h, final) for three hop varieties (Azacca, Idaho-7, and Sultana).

			Azacca Hops			Idaho-7 Hops			Sultana Hops			
Family	Analyte	RT ^2^	6 h	24 h	Final	6 h	24 h	Final	6 h	24 h	Final	Aroma
Monoterpene	β-Pinene	11.4		0.050 ± 0.015	0.017 ± 0.008		0.006 ± 0.001	0.007 ± 0.002	0.029 ± 0.009	0.224 ± 0.067	0.017 ± 0.001	Piney, woody [19]
	β-Myrcene	11.9	0.686 ± 0.201	0.534 ± 0.115	0.601 ± 0.149	0.126 ± 0.064	0.254 ± 0.103	0.085 ± 0.029	0.140 ± 0.042	0.416 ± 0.240	0.661 ± 0.097	Balsamic, woody, herbal [19]
	β-Ocimene	14.6	0.017 ± 0.008	0.012 ± 0.004	0.014 ± 0.003	0.005 ± 0.003	0.017 ± 0.007					Earthy, smokey, green [19]
	Cosmene	18.6			0.011 ± 0.003			0.005 ± 0.002	0.005 ± 0.002	0.018 ± 0.005		
Sesquiterpene	Ylangene	29.6		0.003 ± 0.001	0.007 ± 0.002					0.018 ± 0.005	0.008 ± 0.002	Spicy, fresh, woody [32]
	α-Copaene	29.8	0.004 ± 0.001	0.008 ± 0.002	0.011 ± 0.003		0.006 ± 0.002	0.005 ± 0.002	0.004 ± 0.001	0.025 ± 0.007	0.020 ± 0.004	Woody, wax, honey [19]
	β-Caryophyllene	31.6	0.064 ± 0.020	0.115 ± 0.032	0.152 ± 0.038	0.019 ± 0.001	0.053 ± 0.021	0.054 ± 0.018	0.063 ± 0.019	0.345 ± 0.104	0.319 ± 0.110	Oily, fruity, woody, spicy [19]
	α-Humulene	33.1	0.196 ± 0.061	0.334 ± 0.119	0.413 ± 0.102	0.057 ± 0.027	0.170 ± 0.068	0.130 ± 0.044	0.235 ± 0.071	1.478 ± 0.443	0.875 ± 0.433	Woody, musty [19]
	γ-Muurolene	34.0	0.010 ± 0.003	0.018 ± 0.005	0.024 ± 0.006	0.003 ± 0.001	0.017 ± 0.007	0.009 ± 0.003	0.009 ± 0.003	0.045 ± 0.014	0.031 ± 0.014	
	β-Eudesmene	34.4		0.008 ± 0.002	0.011 ± 0.003			0.006 ± 0.002	0.003 ± 0.001	0.016 ± 0.005	0.011 ± 0.004	
	γ-Gurjunene ^3^	34.7		0.010 ± 0.003	0.014 ± 0.003				0.003 ± 0.001	0.023 ± 0.007	0.017 ± 0.006	Woody [32]
	α-Muurolene	35.0		0.006 ± 0.003	0.009 ± 0.002				0.004 ± 0.001	0.029 ± 0.009	0.009 ± 0.004	
	γ-Cadinene	35.5		0.017 ± 0.005	0.021 ± 0.005	0.014 ± 0.006	0.043 ± 0.017	0.008 ± 0.003	0.009 ± 0.003	0.056 ± 0.017	0.029 ± 0.008	Herbal, thyme, woody [31]
	δ-Cadinene	35.9	0.017 ± 0.006	0.026 ± 0.010	0.033 ± 0.008	0.011 ± 0.005	0.040 ± 0.016	0.015 ± 0.005	0.012 ± 0.004	0.089 ± 0.027	0.032 ± 0.012	Woody [18]
	α-Cadinene	36.4			0.006 ± 0.001					0.015 ± 0.004	0.006 ± 0.002	
	α-Calacorene	36.6			0.010 ± 0.002				0.003 ± 0.001	0.017 ± 0.005	0.005 ± 0.002	Woody [18]
	Cadalene	41.6			0.007 ± 0.002				0.008 ± 0.002	0.065 ± 0.020	0.004 ± 0.001	

^1^ All results are reported as $\bar{x}$ ± s for triplicate experiments. Control (non-hopped) results have been subtracted from these data. From the 6 h sample to the final sample, analytes in red decreased in concentration and analytes in green increased in concentration. Analytes in black did not consistently increase or decrease in all samples. ^2^ Retention time (RT) is reported in min. ^3^ Analyte matched <10% with the NIST database.

**Table 4:** Measurements of specific gravity (SG) and pH and SPME sample collection during fermentation with specific hop varieties.

Hop Variety	Days Since Inoculation + Hopping	SG ^1^	pH ^1^	SPME Samples Analyzed
**Azacca hops**	0	1.057 ± 0.001	5.21 ± 0.04	6 h (Control, T1, T2, T3)
1.6–2.5 mL/100 g total oils	1	1.036 ± 0.002	4.93 ± 0.06	24 h (Control, T1, T2, T3)
14–16% alpha acids	5	1.009 ± 0.001	4.40 ± 0.02	
4–5.5% beta acids	14	1.009 ± 0.001	4.38 ± 0.03	Final (Control, T1, T2, T3)
**Idaho-7 hops**	0	1.059 ± 0.002	5.12 ± 0.05	6 h (Control, T1, T2, T3)
1.0–5.0 mL/100 g total oils	1	1.047 ± 0.014	4.86 ± 0.12	24 h (Control, T1, T2, T3)
9–14% alpha acids	5	1.010 ± 0.001	4.60 ± 0.01	
3.5–9.1% beta acids	14	1.006 ± 0.001	4.32 ± 0.02	Final (Control, T1, T2, T3)
**Sultana hops**	0	1.056 ± 0.001	5.12 ± 0.03	6 h (Control, T1, T2, T3)
2.5–4.0 mL/100 g total oils	1	1.025 ± 0.022	4.86 ± 0.12	24 h (Control, T1, T2, T3)
13–15% alpha acids	5	1.008 ± 0.001	4.53 ± 0.06	
4–5% beta acids	14	1.008 ± 0.001	4.46 ± 0.01	Final (Control, T1, T2, T3)

^1^ All results are reported as $\bar{x}$ ± s for triplicate experiments.

### 2.2. Aroma Development and Hops Biotransformation

To better understand how the natural products from each hop variety were extracted and/or biotransformed by the *S. cerevisiae* yeast during the fermentation process, we compared samples taken 6 h after hopping to samples taken 24 h after hopping and final, single-hopped beer samples (analyzed after fermentation was complete). The control (non-hopped) sample results were subtracted from all trials in each experiment in order to differentiate volatiles originating from the added hops. All in all, a total of 51 VOCs were semi-quantified in these samples: 19 esters (predominantly methyl and ethyl esters), 13 sesquiterpenes, 7 ketones, 7 alcohols, 4 monoterpenes, and 1 volatile acid (gamolenic acid). The detailed, semiquantified results are tabulated and arranged by chemical family in Table 1, Table 2 and Table 3. Analytes that were measured below the limit of quantification (LOQ) are not included. The LOQ is defined as LOQ = x_B_ + 10σ (x_B_ = mean signal of replicate reagent blanks, N = 10; and σ = standard deviation of the blank). In Table 1, Table 2 and Table 3, species that increase in concentration in all samples from the 6 h sample to the fermented beer sample are in green, and those that decrease in concentration are in red.

**Figure 3:**
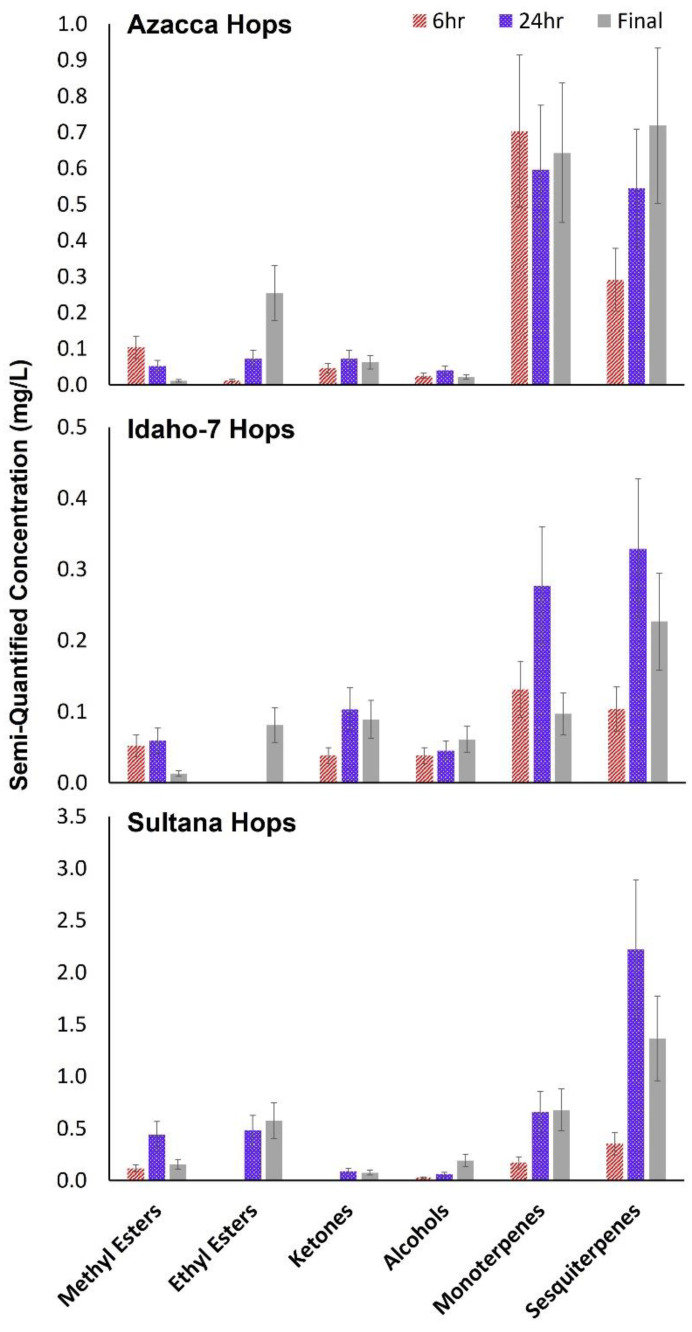
Total concentrations of methyl esters, ethyl esters, ketones, alcohols, monoterpenes, and sesquiterpenes are shown for 3 hop varieties 6 h after hopping, 24 h after hopping, and for the final beer after fermentation was complete.

Esters are both formed and consumed during fermentation in interesting ways. Every methyl ester, with the exception of methyl geranate, decreased in concentration throughout the fermentation process (Table 1). These species typically undergo hydrolysis or transesterification. However, the methyl esters of certain conjugated acids, such as methyl geranate, have been shown to resist hydrolysis during fermentation, explaining the persistence of this methyl ester in the final beer samples [6,14]. Every ethyl ester measured in this study increased in concentration during fermentation (Table 1). Recent work suggests that there are multiple possible causes for this, from yeast enzymatic pathways to transesterification of other hop-derived, precursor esters [33]. The transfer rates of ethyl esters from hops into beer during dry-hopping have been shown to be well over 100%, indicating that these esters are newly formed. However, the exact precursors and processes involved in their formation is still under study. Both 1-methylbutyl propionate and 2-methylbutyl 2-methyl propanoate (Table 1) are possible hop-derived ethyl ester precursors [33]. The observed decrease in methyl ester concentration and increase in ethyl ester concentration from the 6 h samples to the final beer samples is illustrated in Figure 3 for all three hop varieties. The only hop variety that showed similar concentration of methyl esters in the 6 h sample and the fermented beer was Sultana, and this was a result of the increased concentration of methyl geranate in this sample. Pay close attention to the *y*-axis of Figure 3, as it demonstrates the increased overall concentrations of VOCs measured in the headspace of Sultana-hopped beer compared to samples prepared with Azacca or Idaho-7 in these experiments.

Table 2 features the ketones, alcohols, and volatile acids that were semi-quantified in each single-hopped sample. Unsaturated ketones, including 2-nonanone, 2-decanone, 2-undecanone (the most abundant ketone in this study and in others [34]), 2-dodecanone, and 2-tridecanone are well-known constituents of hop oil, as is gamolenic acid [35]. However, other ketones measured in the fermented beer are often produced by yeast according to previous work [34]. These analytes were observed to either increase or plateau in concentration after the 6 h sample was taken, which is a result of both extraction from the hops pellets and yeast production [14]. The monoterpene alcohol linalool was the most abundant alcohol semiquantified in these samples, has the lowest odor detection threshold of the terpene alcohols, and is involved in many biotransformation processes [14]. In addition, linalool has had exceptionally high odor activity values in dry-hopped beers with other hop varieties including Hallertauer Mandarina Bavaria, Hallertauer Cascade, and Hallertauer Mittelfrüh according to recent work [9]. Linalool, as well as cis-geraniol, citronellol, and α-terpineol (Table 2), can all be synthesized by *S. cerevisiae* in varying quantities [34]. These four alcohols are all central to complex enzyme catalyzed biotransformations including cyclizations, isomerizations, reductions, and translocations, as described in previous work [14,34]. Additionally, monoterpene alcohols that were originally glycosidically bound in the raw hops can be released during fermentation, resulting in the overall concentration increase that is observed for several of these analytes (Table 2) [3]. Notably, linalool, cis-geraniol, and citronellol are aroma markers that enhance the esters described previously with more citrus and tropical fruit attributes. The cumulative concentrations of ketones and alcohols in the headspace of each single-hopped beer are summarized in Figure 3. Neither of these chemical classes showed consistent trends over fermentation time across the hop varieties, evidencing a difference in concentration of these hop-derived species in the Azacca, Idaho-7, and Sultana hop oil as well as complex involvement in biotransformations (specifically with respect to the monoterpene alcohols) [36]. 

Measured concentrations of volatile monoterpenes and sesquiterpenes in the 6 h, 24 h, and final single-hopped samples are detailed in Table 3 and summarized cumulatively in Figure 3. The most abundant terpenes of these hop essential oils (β-myrcene, α-humulene, and β-caryophyllene) were observed in relatively high concentrations in the final products, despite their well-known adsorption at the hydrophobic yeast cells and migration to the foam layer [14]. Β-Myrcene is the most abundant known hop oil constituent (typically >80% *v*/*w* in the hops trichome) [3], and both α-humulene and β-caryophyllene are abundant in hops leaves and flowers. In Figure 3, overall monoterpene concentrations are shown to either increase from the 6 h sample to the final sample (Sultana) or stay the same within error (Azacca and Idaho-7), whereas the sesquiterpene concentrations increased from the 6 h to the final beer, regardless of the hop variety used. These species are simply extracted natural products originating from the hop oils that do not undergo subsequent biotransformations or chemical alterations [14]. Indeed, current reviews of the literature indicate that yeast cannot biotransform compounds from these chemical families during dry hopping [34]. Notably, these volatile monoterpene and sesquiterpene concentrations will vary considerably depending on the timing of dry hopping (during or after fermentation). Further study is necessary to elucidate this in detail.

### 2.3. Sensory Analysis

**Figure 4:**
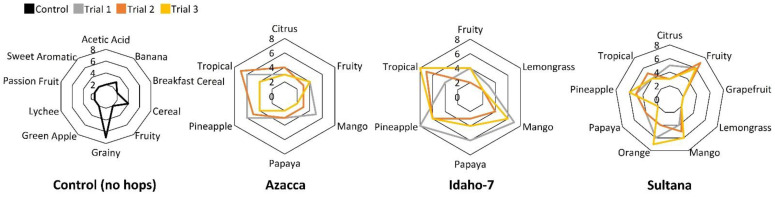
Sensory analysis results using the CATA method on the DraughtLab Pro App are shown here for a control, non-hopped sample (black) and three single-hopped samples hopped with Azacca, Idaho-7, and Sultana each. Hopped sample sensory analyses were conducted in triplicate, wherein each trial was fermented separately (gray, orange, and yellow). Axes numbers represent the number of panelists reporting this aroma.

The results of the sensory panel, shown in Figure 4, complement these chemical analyses and are directional in nature. Because these results are directional, and the number of participants is relatively small, comprehensive statistical analysis was not conducted here. Rather than drawing statistical conclusions from these data, we sought to preliminarily understand which hops a consumer (untrained panelist) might prefer and how single hopped beers prepared with different hop varieties are perceived differently by those panelists. Untrained, volunteer panelists were given a carbonated beer sample and a list of aroma options and asked to ‘choose all that apply’ (CATA) using the DraughtLab Pro App to describe the aroma of each sample. The choices available to the participants are included in Supplementary Materials (Table S1). Participants identified primarily grainy, fruity, and cereal aromas from the control sample (shown in black), which lacked hops. Some of the sweet, fruit, and banana aromas that were also selected in the control sample are likely the result of the ethyl esters (including ethyl octanoate and ethyl decanoate) that were measured in the control sample headspace at lower abundance than the hopped samples. Three trials were conducted for each single-hopped beer, and the spread in results across these trials is not surprising given the fact that the panelists were untrained. These are shown in Figure 4 as trial 1 (gray), trial 2 (orange), and trial 3 (yellow). The aromas selected for the control (non-hopped) sample (cereal, grainy, etc.) compared to the hopped samples (tropical, mango, etc.) in Figure 4 highlight the fundamental importance of hops addition to produce an appealing aroma.

Several striking similarities between the hop varieties can be observed in Figure 4. For example, five of the reported aromas are shared across all three single-hopped beers: tropical, fruity, pineapple, papaya, and mango. These sweet, fruity aromas were likely produced by the mixture of ethyl and methyl esters that were measured in all three beer samples including ethyl octanoate, ethyl nonanoate, ethyl trans-4-decenoate, ethyl decanoate, and methyl geranate. One aroma, citrus, is only identified by panelists in Azacca and Sultana-hopped beers. Humulenol II, a VOC that was only measured in Azacca and Sultana-hopped samples, has a citrus aroma [31] and may be a contributor to this sensory panel identification. Similarly, lemongrass aroma was identified by the sensory panel in only Idaho-7 and Sultana-hopped samples. Jasmine lactone and citronellol are both known to have sweet, floral aromas [24,28] and are unique to only Idaho-7- and Sultana-hopped samples. Linalool is also a VOC with a sweet, floral aroma, and this analyte was semi-quantified in the top ten most abundant analytes for both Idaho-7- and Sultana-hopped samples, as shown in Figure 2. Therefore, many of these sensory impressions (orange, citrus, mango, and pineapple) can be linked to esters and terpene alcohols, which were measured in this study. It is important to note that thiols, which are not measurable by the analytical methods described herein [6], may also synergistically contribute to these sensory attributes at low concentrations [5].

There are notable differences among the hop varieties as well. A greater quantity of unique aromas (nine) was reported for Sultana-hopped beer than for Azacca- or Idaho-7-hopped beers (six each), corroborating the chemical analysis result that a greater variety of unique, volatile analytes were measured in Sultana (39) compared to Azacca (35) or Idaho-7 (28) (see Table 2, Table 3 and Table 4). Based on the top ten analytes in Sultana-hopped beer (see Figure 2), it is no surprise that the fruity aroma stood out to panelists. There was a wider variety of fruity esters and a greater overall concentration of esters in the Sultana sample than in the other hopped beers. Although there was great similarity between Idaho-7- and Sultana-hopped samples, the greatest difference between them was in the increased fruity notes imparted by Sultana. Two aromas chosen by the sensory panelists, grapefruit and orange, were reported only for Sultana-hopped samples. These are the only two aromas in this study that were unique to one sample type. Although there are several possible analytes responsible for these identified grapefruit and orange aromas, cis-geraniol (orange, citrus) stands out as one analyte that was only measured in the Sultana-hopped beer and is described in the literature to match this aroma well [19]. Previous publications have highlighted the grapefruit notes in other hop varieties including Kazbek and Chinook, both of which have cis-geraniol as a dominant aromatic compound [8]. Notably, despite the large overall concentration (see Figure 3) of monoterpenes and sesquiterpenes with woody, spicy aromas, these options were not selected by the sensory panelists. The fruity, sweet esters, alcohols, and ketones dominate the aroma profile over these terpenic VOCs for all three hop varieties studied. Whether these woody, spicy aroma attributes are perceptible varies greatly with hop variety, according to recent work [10]. Spicy notes are dominant in many other hop varieties including Kazbek, Saaz, Vital, Spalter Select, Tettnang, and Tettnanger [10]. Lastly, it is important to note that perception thresholds drive sensory aroma perception, and those thresholds for many of the analytes measured herein have been previously published in the literature [20,37].

## 3. Materials and Methods

### 3.1. Fermentation

Four 3.5 barrel portions of unhopped Hazy IPA wort (Ska Brewing Co.) were diverted from a single 30 barrel batch and transferred into four 7 barrel conical fermenters. This allowed the experiments to be conducted in triplicate with one control (non-hopped) fermenter. Hazy IPA wort was prepared in a single infusion mash tun with a mash temperature of 68 °C and a 30 min mash rest. The grist bill was comprised of the following: 40% two-row malt, 15% malted wheat, 17% dextrin malt, 7% golden naked oats, 15% flaked oats, and 5% flaked barley. There were no kettle additions. Fermentis SafAle S-O4 dehydrated *Saccharomyces cerevisiae* yeast (250 g) was added to each fermenter. At the same time as inoculation, 10.5 pounds of T90 hop pellets were added to fermenters 1, 2, and 3. No hop pellets were added to fermenter 4 (control). The yeast was fully interactive with the hops during the entirety of fermentation. All fermenters were set to 21.2 °C for the duration of the fermentation. This experiment was conducted separately for each single hop variety: Azacca (Crosby Hop Farms, Woodburn, OR, USA), Idaho-7 (Crosby Hop Farms, Woodburn, OR, USA), and Sultana hops (Hopsteiner, New York, NY, USA). Details reported by the suppliers including total oil, alpha acids, and beta acids for each hop variety are included in Table 4. 

Samples were taken for analysis 6 h and 24 h after yeast and hops were added. When sampling, the sample port was sterilized with isopropyl alcohol and a propane blow torch. Samples were taken directly from the sample port into a 50 mL vial, filled to the brim to limit oxygen interaction. Samples were then transported to the lab for immediate analysis. Specific gravity (SG) was measured using an Anton Paar DMA35 handheld density meter, and pH was measured using a Vernier pH probe. After final gravity was reached, diacetyl (2,3-butanedione) levels were measured using GC headspace analysis (ASBC method Beer 25F-B) [38]. Once diacetyl levels in all 4 tanks reached less than 60 ppb, the tanks were cooled to −0.56 °C simultaneously. Three days after cooling, the final sample was taken for analysis. At this time, sensory samples were also taken from each tank. Table 4 outlines the timeline of the three single-hop experiments, including the SG and pH readings and the times when SPME samples were taken for analysis. 

### 3.2. Sensory Panel

To measure the perceived similarities and differences between the three single hop beer aromas, 1 L samples were taken by hand from each fermenter 72 h after cooling. Since each dry-hopped sample was prepared separately in time, the control, T1, T2, and T3 prepared with one hop variety were introduced to the sensory panel during each session. Three separate sensory data collection sessions were held to collect the same results from each of the three dry-hopped beers prepared in triplicate. To carbonate the samples, each was attached to a pure CO_2_ source while in a sealed bottle. All sensory data were collected the same day of sample collection, and samples were stored in containers with minimal headspace to limit oxygen exposure to the samples. The samples from each of the fermentation vessels were presented blind and in random order to volunteer, untrained participants at Ska Brewing Company (IRB-2021-176 Exempt Determination). Between 14 and 22 panelists (as many as were available at the time point required for analysis) participated in each of the trials. Panelists were asked to describe each of the four samples using the Draughtlab Pro sensory app description builder through a check-all-that-apply (CATA) method [20,39]. Supplemental Materials associated with this publication includes every option available for sensory panelists to choose from (Table S1).

### 3.3. Solid Phase Microextraction (SPME)

Beer samples were prepared for SPME in 20 mL 22.5 × 75 mm glass headspace vials (Sigma Aldrich, Cleveland, OH, USA). A 10 mL sample aliquot was combined with 2.6 g of NaCl for salting out and 1 μL of 877 mg L^−1^ methyl octanoate-d15 internal standard (C/D/N Isotopes Inc., Pointe-Claire, QC, Canada, 98.7%) prepared in ethyl acetate (Sigma Aldrich, USA). Therefore, each 10 mL sample contained 0.087 ± 0.002 mg L^−1^ of internal standard prior to SPME. Each vial was capped with a 20 mm PTFE/silicone liner cap (Sigma Aldrich, USA) and parafilm. The vials were partially immersed in a 40 ± 2 °C water bath for 10 min to allow for headspace equilibration prior to SPME fiber introduction. A 50/30 μm DVB/CAR/PDMS SPME fiber (Millipore Sigma, USA, 24 ga needle size) was introduced into the headspace of each sample vial and held there for 30 min. SPME fibers were stored at 4.4 °C until analysis by GC-MS (within 24 h of exposure). SPME fibers were conditioned after every GC injection for 30 min at 270 °C before being exposed to a new sample headspace.

### 3.4. Gas Chromatography–Mass Spectrometry Analysis

An Agilent 7820A Gas Chromatograph (GC) with an HP-5MS 30 m × 0.25 mm × 0.25 μm column and a 5977E Mass Spectrometer Detector (MSD) with MassHunter GC-MS Acquisition Software (B.07.00.1203 Agilent, Santa Clara, CA, USA) was used to separate and detect sample VOCs. During the splitless injection, SPME fibers were manually held in the GC inlet for 1 min at 260 °C to ensure all VOCs had desorbed. Helium carrier gas flow was set to 1 mL min^−1^ for the duration of the separation. The oven temperature was held at 50 °C for 5 min, increased first to 190 °C (3 °C min^−1^), then increased to 230 °C (70 °C min^−1^), and then held constant at 230 °C for 5 min. The transfer line leading to the mass spectrometer was held at 280 °C. An electron ionization (EI) source (70 eV), a single quadrupole (*m*/*z* 50–550), and an electron multiplier were used for VOC detection and identification. Consistent with previous work [22,23], siloxanes originating from the GC column and the DVB/CAR/PDMS fiber were routinely observed, but these species did not interfere with analyte identification and semi-quantification. The most recently released National Institute of Standards and Technology (NIST) library (2020, Agilent Technologies) was used to identify analytes by their EI fragmentation patterns. Analytes were semi-quantified based on the ratio between their integrated peak area and the integrated peak area of the methyl octanoate-d15 internal standard [20].

## 4. Conclusions

Based on current knowledge of hop varieties and the biotransformation of their oils, it remains challenging to predict the impact of hops addition on the aroma of dry-hopped beer [3]. This is due to the complexity of hop oil extraction and biotransformation processes as well as the lack of published chemical and sensory analysis of beer prepared with various hop varieties using specific hopping methods. Through the combined chemical and sensory analysis of three single-hopped beers dry-hopped with Azacca, Idaho-7, and Sultana hops, the results reported herein begin to address this challenge. First of all, the results of chemical analyses via HS-SPME-GC-MS are in agreement with directional sensory panel results. Dry hopping with Sultana yielded a final beer with a larger quantity of unique, semi-quantified VOCs (39); a higher overall concentration of headspace VOCs (3.2 mg L^–1^); and a larger array of unique aromas identified by sensory panelists (nine) than beer prepared with either Azacca (35 VOCs, 1.7 mg L^–1^, six aromas identified) or Idaho-7 (28 VOCs, 0.6 mg L^–1^, six aromas identified). From an efficiency standpoint, this is an important observation: the same quantity of Sultana hops delivers a measurably increased concentration of aroma-active species in the headspace compared to Azacca or Idaho-7, according to these results. Those aromas that exist above perception thresholds [37] drive the sensory experiences of consumers. There are also notable similarities between dry-hopped beers prepared with these three hop varieties: seven of the top ten most abundant analytes are the same for all three single-hopped beers, for example, although they vary in abundance (β-myrcene, 2-methylbutyl 2-methylpropoanoate, ethyl octanoate, ethyl trans-4-decenoate, ethyl decanoate, β-caryophyllene, and α-humulene). The sensory panel results corroborate this observation as well. Five of the six total aromas that were selected by panelists in Azacca- and Idaho-7-hopped beer and five of the nine total aromas identified in Sultana-hopped beer were shared among all three beers (tropical, fruity, pineapple, papaya, and mango). At three points (6 h, 24 h, and 14 days) after simultaneous inoculation and dry hopping, the aroma of each beer was profiled to better understand and observe aroma development during fermentation. Methyl esters (with the exception of methyl geranate) decreased in concentration over time, likely due to hydrolysis or transesterification. Ethyl esters increased in concentration over time, as a result of transesterification and yeast production. Ketones, alcohols, and volatile acids all increased in concentration or showed mixed trends across the hop varieties due to a combination of their various natural abundances in each hop oil as well as their subsequent involvement in previously published biotransformations [34,35]. Lastly, a diverse array of monoterpene and sesquiterpene species were successfully extracted into the beer from the hops pellets but were not subsequently biotransformed by the yeast [34]. These terpenic woody, spicy aromas were notably not selected by the sensory panelists, highlighting the one difference between the chemical and sensory results of this work, as well as the importance of volatile headspace concentration and perception threshold. 

Overall, through the study of different hop varieties and the extraction as well as biotransformation of their oils, the brewing process can be increasingly optimized. All other variables held constant, the same quantity of Sultana hops delivers a higher array and concentration of unique volatiles that are identifiable through chemical and sensory methods than Azacca or Idaho-7 hops through this method of dry hopping. Furthermore, sensory panelists identified two unique aromas, grapefruit and orange, exclusively in Sultana-hopped beer. Based on these data, Sultana hops is an ideal choice for imparting a complex, citrus and fruity aroma to dry-hopped beer while conserving this expensive raw ingredient. To elucidate the connection between hop variety and hopping method on beer aroma, future work is necessary to continue profiling the aromas and biotransformation processes taking place during the fermentation of hopped beers.

## Data Availability

Not applicable.

## Supplementary Materials

The following supporting information can be downloaded at https://www.mdpi.com/article/10.3390/molecules28155802/s1, Table S1: Aroma ‘Check All That Apply’ (CATA) Options in Draught Lab Pro App.
