# Supplementary material for: Aroma Profile Development in Beer Fermented with Azacca, Idaho-7, and Sultana Hops
Source: Molecules. 2023 Aug 1;28(15):5802. doi: 10.3390/molecules28155802 (PMC10421000; doi:10.3390/molecules28155802)
Supplement: Supplementary file 1 [file molecules-28-05802-s001.zip › molecules-2509764-supplementary.pdf]

Supplementary Information

Table S1. Aroma 'Check All That Apply' (CATA) Options in Draught Lab Pro App

First Selection	Second Selection	Third Selection
Chemical	2-Acetyl Pyridine (Biscuit), 4-Ethyl Guaiacol (Medicinal, Spicy, Clove, Burnt, Smoke, Burnt Rubber), 4-Ethyl Phenol (medicinal, band-aid, earthy, barnyard, plastic), 4-Vinyl Guaiacol (spicy, sweet aromatic, clove, burnt, smoke, vanilla), Acetaldehyde (vegetal, paint, green leaves, green apple, grassy, solvent, fruity), Acetic Acid (vinegar, solvent), Acetylpyridine (cereal, biscuit, popcorn), Benzaldehyde (Marzipan, Cherry, Woody, Sweet Aromatic, Burnt Sugar, Nutty, Almond), Butyric Acid (dairy, sewage, sweat, butter, cheese, fruity), Caprylic Acid (waxy, fat, vegetable oil), Catty (Back currant/catty, fruity), Chlorophenol (medicinal, antiseptic), cis-3-hexanal (green leaves, grassy, watermelon, fresh cut grass, fruity), cis-3-hexanol (waxy, banana, cucumber, vegetal, green leaves, grassy, fresh cut grass, fruity), citric acid (sour), citronellol (lychee, rose, citronella, lime), damascenone (dried fruit, woody, sweet aromatic, stewed apple, strawberry rhubarb, herbaceous, black currant/catty, raspberry, peach, earthy, dry leaves, solvent, tobacco, menthol, honey, fruity, cooked fruit), diacetyl (butterscotch, dairy, butter, buttermilk, earthy, mold), dimethyl sulfide (vegetal, onion, tomato paste, canned corn, baked beans, cooked cabbage), ethyl acetate (solvent, fruity), ethyl butyrate (sweet aromatic, pineapple, bubble gum, fruity, artificial fruit), ethyl hexanoate (waxy, fat, licorice, spicy, green apple, solvent, fruity), geosmin (vegetal, soil, earthy), geraniol (rose, lime, geranium, lemon, floral, fruity, hyacinth), Guaiacol (smoke), Hydrogen sulfide (boiled egg), indole (fecal, cheese), isoamyl acetate (banana, solvent, fruity), isovaleric acid (dairy, cheese), kerosine (geranium, floral, petroleum, solvent), lactic acid (sour), limonene (orange, grassy), linalool (orange, grassy), mercaptan (boiled egg, vegetal, drains), methional (vegetal, cooked potato), myrcene (medicinal, resinous, woody, spicy, vegetal, geranium, herbaceous, pine, lemon, floral, grassy, celery, carrot, plastic, fruity, balsamic, black pepper), styrene (medicinal, plastic), sulfur dioxide (struck match, burnt), trans-2-nonenal (stale, waxy, fat, tallow, cardboard, cucumber, vegetal, paper), trichloroanisol (earthy, musty, mold), vanillin (sweet aromatic, vanilla)	N/A
Fruity	Citrus	Grapefruit, orange, lemon, lime
	Tropical	Mango, pineapple, papaya, banana, lychee, guava, passionfruit, coconut
	Stone Fruit	Peach, apricot, nectarine, cherry, plum

	Berry<br><br><br><br><br><br><br><br>Pomme<br><br><br>Melon<br><br><br><br><br>Dried Fruit	Raspberry, Strawberry, Blueberry, Blackberry, Concord grape, Muscat grape, Gooseberry, Black Currant, Red Currant<br><br>Green apple, red apple, pear, cider<br><br>Cantaloupe, honeydew, cucumber, watermelon<br><br>Prune, fig, date, raisin
Floral	Geranium, rose, citronella, lavender, lilac, chamomile, honeysuckle, jasmine, lily, violet, perfume	N/A
Sweet Aromatic	Caramel, honey, toffee, chocolate, brown sugar, burnt sugar, molasses, vanilla, bubble gum, frosting, marshmallow, pie crust, maple syrup, cola	N/A
Spicy	Black pepper, white pepper, nutmeg, allspice, clove, juniper, licorice, coriander, cinnamon, ginger, caraway	N/A
Herbaceous	Black tea, green tea, mint, rosemary, dill, thyme	N/A
Grassy	Fresh cut grass, lemongrass, green leaves, hay, dry leaves	N/A
Earthy	Musty, soil, barnyard, marsh/moss, mushroom, beet	N/A
Woody	Pine, resinous, cedar, tobacco, sandalwood, eucalyptus, spruce, oak	N/A
Nutty	Walnut, almond, peanut, hazelnut	N/A
Cereal	Grainy, white bread, white cracker, bread dough, graham cracker, breakfast cereal, biscuit, toast, corn chip	N/A
Burnt	Coffee, smoke, struck match	N/A
Stale	Leather, paper, cardboard, waxy, goat hair, wet dog, meaty/soy sauce, mousy	N/A
Dairy	Butter, butterscotch, sour milk, cheese, yogurt	N/A
Rotten	Sweat, boiled egg, rotten garbage, fecal, baby vomit, rotten cheese, fish, lightstruck/skunky, rotten vegetable	N/A
Vegetal	Onion, green onion, fried onion, green bell pepper, garlic, canned corn, tomato paste, cooked cabbage, celery, carrot, cooked potato, baked beans, black olive, cooked vegetable	N/A
Medicinal	Band-aid, plastic, shower curtain, antiseptic, garden hose, cough syrup	N/A
Solvent	Vinegar, paint thinner, nail polish remover, petroleum/diesel	N/A
Alcoholic	Sherry, red wine, white wine, rum, whiskey	N/A
Metallic	Metallic	N/A
